# Exosomes derived from gefitinib-treated EGFR-mutant lung cancer cells alter cisplatin sensitivity via up-regulating autophagy

**DOI:** 10.18632/oncotarget.8358

**Published:** 2016-03-25

**Authors:** Xiao-Qiu Li, Jia-Tao Liu, Lu-Lu Fan, Yu Liu, Liang Cheng, Fang Wang, Han-Qing Yu, Jian Gao, Wei Wei, Hua Wang, Guo-Ping Sun

**Affiliations:** ^1^ Department of Oncology, The First Affiliated Hospital of Anhui Medical University, Hefei, Anhui, China; ^2^ Institute of Clinical Pharmacology, Anhui Medical University, Hefei, Anhui, China; ^3^ Department of Pharmacy, The First Affiliated Hospital of Anhui Medical University, Hefei, Anhui, China

**Keywords:** lung cancer, exosomes, gefitinib, cisplatin, autophagy

## Abstract

Several clinical trials indicate that concurrent administration of tyrosine kinase inhibitors (TKIs, such as gefitinib or erlotinib) with chemotherapy agents fails to improve overall survival in advanced non-small cell lung cancer (NSCLC) patients. However, the precise mechanisms underlying the antagonistic effects remain unclear. In the present study, we investigated the role of exosomes in the antagonistic effects of concurrent administration of chemotherapy and TKIs. Exosomes derived from gefitinib-treated PC9 cells (Exo-GF) decreased the antitumor effects of cisplatin, while exosomes derived from cisplatin-treated PC9 cells (Exo-DDP) did not significantly affect the antitumor effects of gefitinib. Additionally, inhibition of exosome secretion by GW4869 resulted in a modest synergistic effect when cisplatin and gefitinib were co-administered. Furthermore, Exo-GF co-incubation with cisplatin increased autophagic activity and reduced apoptosis, as demonstrated by an upregulation of LC3-II and Bcl-2 protein levels and downregulation of p62 and Bax protein levels. Thus, the antagonistic effects of gefitinib and cisplatin were mainly attributed to Exo-GF, which resulted in upregulated autophagy and increased cisplatin resistance. These results suggest that inhibition of exosome secretion may be a helpful strategy to overcome the antagonistic effects when TKIs and chemotherapeutic agents are co-administered. Before administering chemotherapy, introducing a washout period to completely eliminate TKI-related exosomes, may be a better procedure for administering chemotherapy and TKIs.

## INTRODUCTION

Gefitinib (ZD1839, Iressa) is a widely used treatment for NSCLC patients who harbor the EGFR mutation. Several randomized phase III clinical trials have demonstrated that the simultaneous administration of gefitinib or erlotinib with platinum-based doublet chemotherapy did not improve overall survival (OS) when compared to chemotherapy alone [1–4]. Although adverse events occurred mainly in the groups that were administered the drug combination, most of the adverse events resulted from the known toxicities of the chemotherapy agents [1–4]. Previous reports indicate that sequential administration of chemotherapy followed by TKIs results in progression-free survival (PFS) when compared to chemotherapy alone [5, 6]. The potential reason for the antagonism between TKIs and chemotherapeutic agents used concurrently is that TKIs can induce cell cycle arrest in the G1-phase, [7, 8]. However, in the FASTACT [5] and FASTACT-2 [9] clinical trials, an interaction between TKIs and chemotherapy was observed even though chemotherapy was administered at the end of erlotinib treatment. Thus, an alternate mechanism may be responsible for the antagonistic effect between chemotherapy and EGFR-TKIs.

Exosomes are small vesicles that originate from endocytic multivesicular compartments and are secreted by a variety of cell types. Additionally, exosomes mediate cell-to-cell or cell-to-environment communication [10, 11]. According to a recent study, exosomes can modulate immune function, angiogenesis and cell proliferation, as well as tumor cell invasion and metastasis [12–14]. Furthermore, exosomes can also change the sensitivities of a recipient cell to some antitumor agents; however, the underlying mechanisms are not fully understood [15, 16].

Autophagy is a highly conserved biological phenomenon in eukaryotic cells [17], but the specific role of autophagy in anticancer therapy is not clear. We, as well as others, have previously reported that gefitinib or chemotherapy agents (e.g., cisplatin) can induce cytoprotective autophagy [18, 19]. However, the impact of exosomes derived from TKIs or chemotherapy agents on autophagy and the sensitivity of recipient cells to other agents are still unknown.

The mechanisms underlying the antagonistic effects of combining TKIs and chemotherapy agents and how tumor cells transmit autophagic signals to un-affected cells are still unknown. In the present study, the role of exosomes in the antagonistic effects of combining chemotherapy and TKIs was investigated using exosomes derived from gefitinib-treated PC9 cells (Exo-GF) and co-incubating these exosomes with cisplatin. Additionally, exosomes that were derived from cisplatin-treated PC9 cells (Exo-DDP) were co-incubated with gefitinib. Our results indicated that Exo-GF significantly decreased the antitumor effects of cisplatin by increasing autophagic activity.

## RESULTS

### Gefitinib combination with cisplatin leads to an antagonistic effect

**Figure 1:**
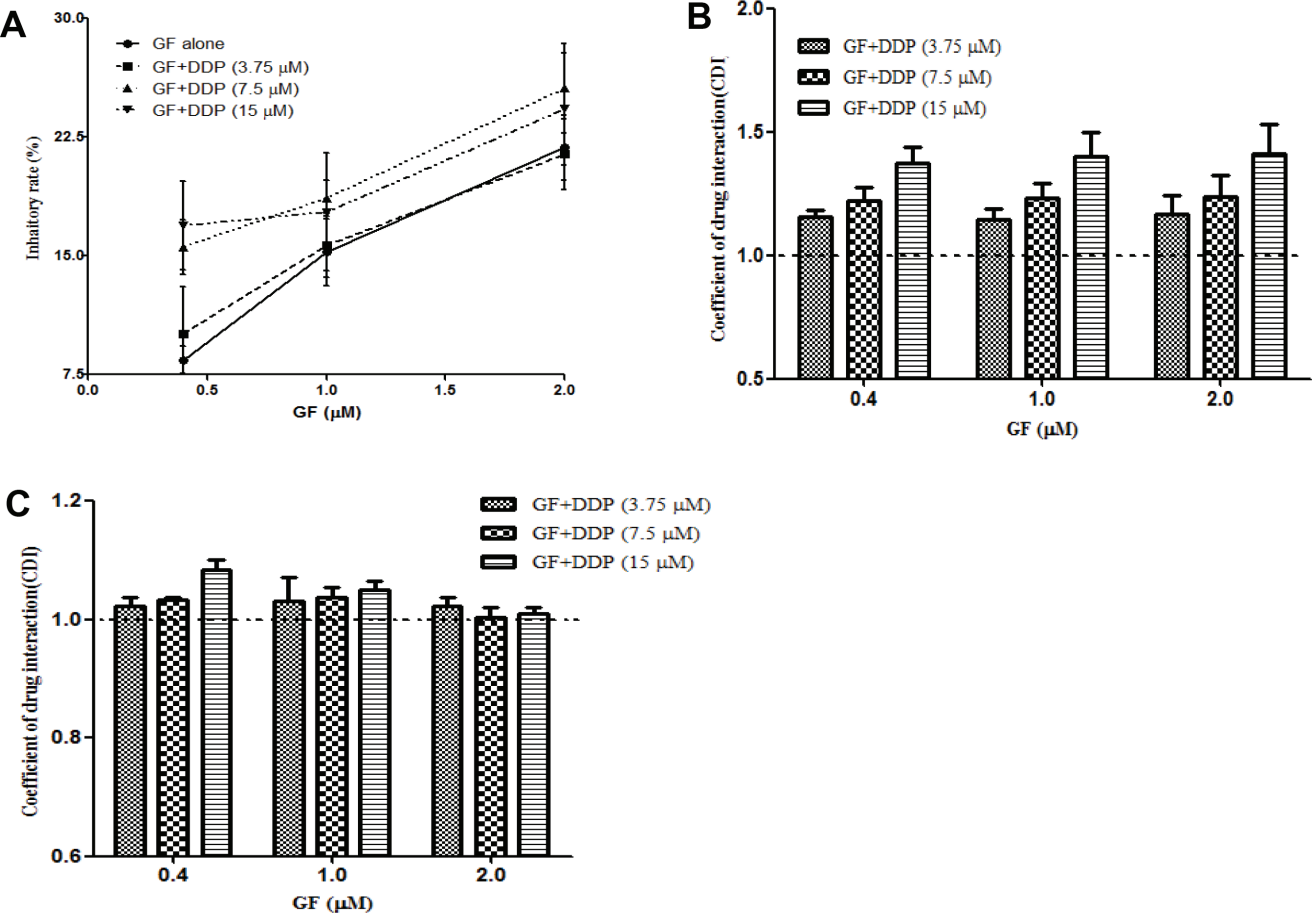
Co-administration of gefitinib (GF) and cisplatin (DDP) result in an antagonistic effect Cells were treated with the indicated concentrations of gefitinib and cisplatin for 24 hours, and the inhibitory ratio of combination groups for PC9 cells (**A**) was measured by CCK-8. CDI values were measured in PC9 cells (**B**) and A549 cells (**C**). CDI < 0.95, CDI > 1.05 and between 0.95 and 1.05 represent synergism, antagonism and additive effects, respectively. Data are presented as the mean ± SD of at least three independent experiments.

To address the nature of the interaction between gefitinib and cisplatin, we conducted the CCK-8 assay using the indicated concentrations of gefitinib and cisplatin. As shown in Figure 1A, combining gefitinib with cisplatin slightly increased the anti-proliferation effects of gefitinib compared to the various concentrations of gefitinib (0.4–2 μΜ) or DDP (the inhibition ratio was 7.5% ± 0.9%, 11.7% ± 2.9% and 22.9% ± 0.7% for 3.75, 7.5 and 15 μM, respectively) treatment alone. The CDI values of 1.16 ± 0.1 (range, 1.04–1.39; Figure 1B) indicate an antagonistic interaction between gefitinib and cisplatin in the EGFR-mutant NSCLC PC9 cell lines. Subsequently, A549, an EGFR wildtype NSCLC cell line, was used to confirm this phenomenon, and a modest antagonistic effect was observed (Figure 1C). These results were consistent with our previous study and the results of others [19–21].

### Characterization of exosomes released by PC9 cells

**Figure 2:**
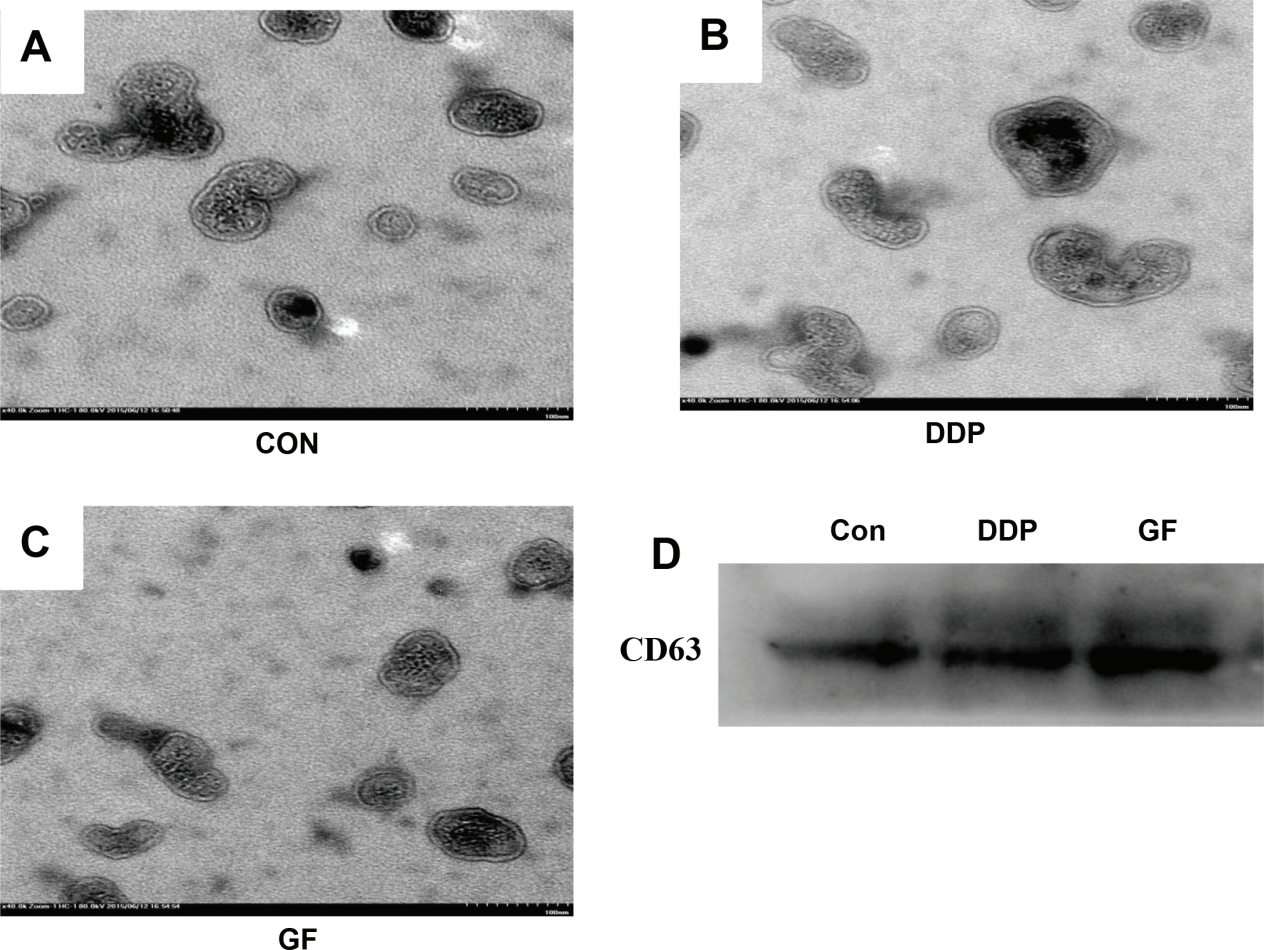
Characterization of exosomes isolated from supernatant samples Morphological characterization of exosomes derived from supernatant samples of PC9 cells from the control cultured group (**A**), 7.5 μΜ DDP group (**B**) and 1 μΜ gefitinib group (**C**), Bar, 100 nm. (**D**) CD63 expression in exosomes isolated from supernatant samples was assessed by western blot analysis.

To ensure that the isolated pellets were genuine exosomes, the collected pellet was captured under a transmission electron microscope (TEM) and analyzed by western blotting. Representative TEM images of exosomes obtained from the supernatant of PC9 cells under different conditions (untreated control, 1 μΜ gefitinib or 7.5 μΜ cisplatin) are shown in Figure 2A–2C. A homogeneous population of round vesicles 40–100 nm in diameter was observed. CD63, a member of the tetraspanin family, is an evolutionarily conserved protein in exosomes and a widely used biomarker for testing exosomes [22]. Western blotting was performed to further confirm that the collected pellets were exosomes by detecting the presence of CD63 in all three samples derived from PC9 cells that underwent different treatments (Figure 2D). We also tested the impact of DDP and GF on the secretion of exosomes using a BCA protein assay kit. It was observed that GF (84.5 ± 9.5 μg/10^7^ cells) significantly increased exosome secretion, while DDP (49.2 ± 15.2 μg/10^7^ cells) had almost no influence on exosome secretion in PC9 cells (50.5 ± 14.9 μg/10^7^ cells).

### Exo-GF decreases the antitumor activity of cisplatin

**Figure 3:**
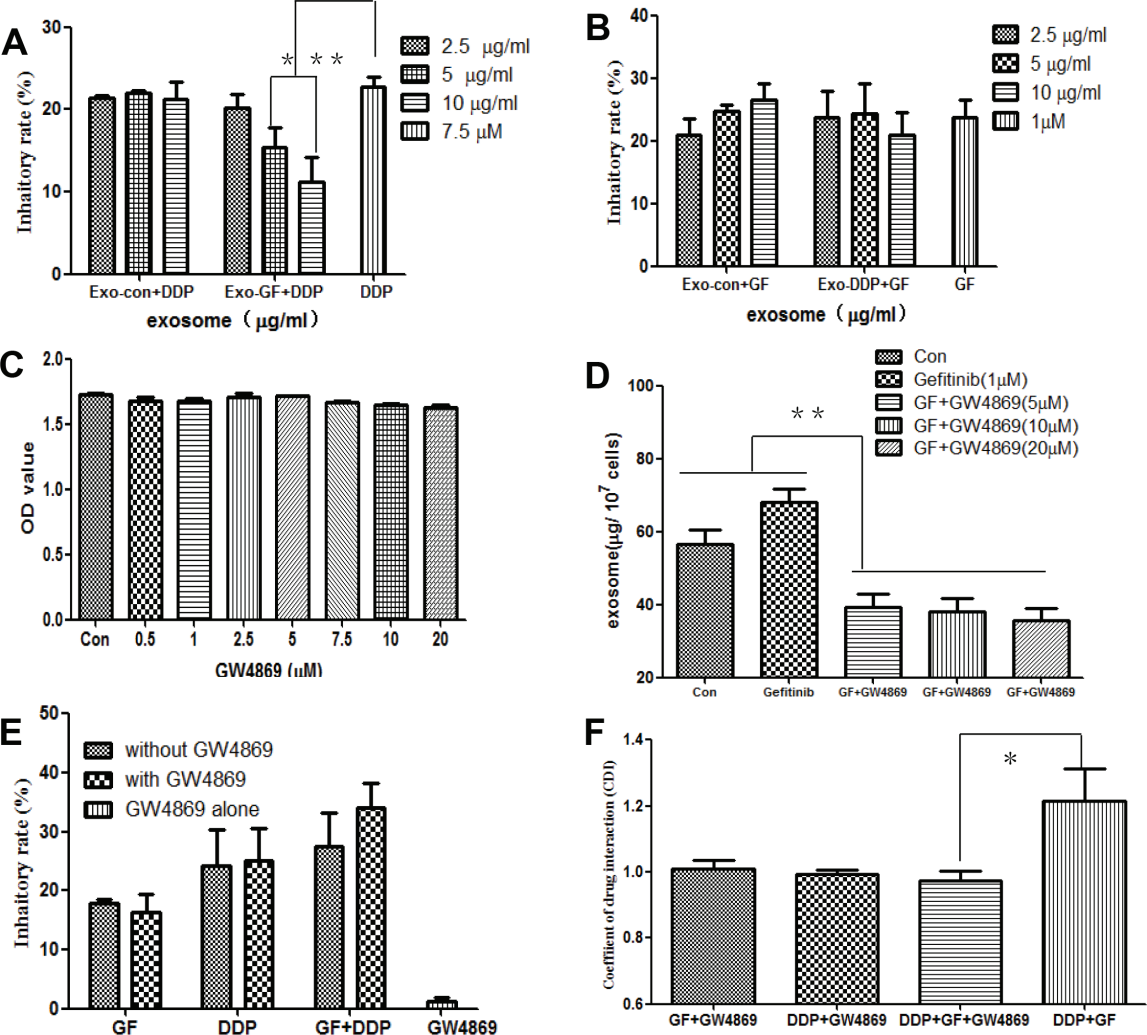
Inhibition of exosome secretion by GW4869 overcomes the antagonistic effects of gefitinib and cisplatin PC9 cells were pre-incubated with the indicated concentrations of Exo-GF or Exo-DDP for 24 hours, and the inhibition of proliferation in cells co-cultured with DDP (**A**) or gefitinib (**B**) was measured by CCK-8 assay. The effects of GW4869 on cell vitality were measured by CCK-8 assay (**C**), and the effect of GW4869 on exosome secretion was measured by BCA (**D**). The anti-proliferation effect of GF and/or DDP in the presence or absence of 10 μM GW4869 was measured by MST (**E**), and CDI values were also analyzed (**F**). Data are presented as the mean ± SD (error bar) of at least three independent experiments. *and **represent *P* < 0.05 and *P* < 0.01, respectively.

To determine whether the antagonistic effects between gefitinib and cisplatin were mediated by Exo-GF or Exo-DDP, exosomes were harvested and added to cells in combination with cisplatin or gefitinib. As shown in Figure 3A, Exo-GF counteracted the antitumor effects of cisplatin in a dose-dependent manner (*P* < .05 and < .01 vs. cisplatin alone in 5 μg/ml exosomes and 10 μg/ml exosomes). Exo-Con did not show any effects on cisplatin-induced growth inhibition. Although a slight neutralization was seen at the highest dose group, Exo-DDP had no effect on gefitinib (Figure 3B).

Next, we investigated whether inhibition of exosome secretion could overcome the antagonistic effects of gefitinib and cisplatin. The administration of GW4869 between 0.5 μΜ and 20 μΜ did not have a significant influence on PC9 cell growth (Figure 3C), but when GW4869 was co-cultured 1 hour before the introduction of gefitinib, there was a significant decrease in exosome secretion (*P* < .01 vs untreated control and gefitinib group), as indicated in Figure 3D. The administration of 10 μΜ GW4869 modestly increased the growth inhibition rate of gefitinib and cisplatin but had little impact on gefitinib- or cisplatin-induced growth inhibition (Figure 3E). CDI values were used to evaluate the nature of the GW4869 interaction with gefitinib and/or cisplatin. As shown in Figure 3F, co-administration of gefitinib or cisplatin with GW4869 produced additive effects, with CDI values of 1.01 ± 0.05 and 1.02 ± 0.02 for gefitinib and cisplatin groups, respectively. The CDI values of GW4869 combined with the co-administration of gefitinib and cisplatin was 0.97 ± 0.05, which indicated a modest synergistic effect.

### Enhanced autophagy contributes to the increased cisplatin resistance by Exo-GF

**Figure 4:**
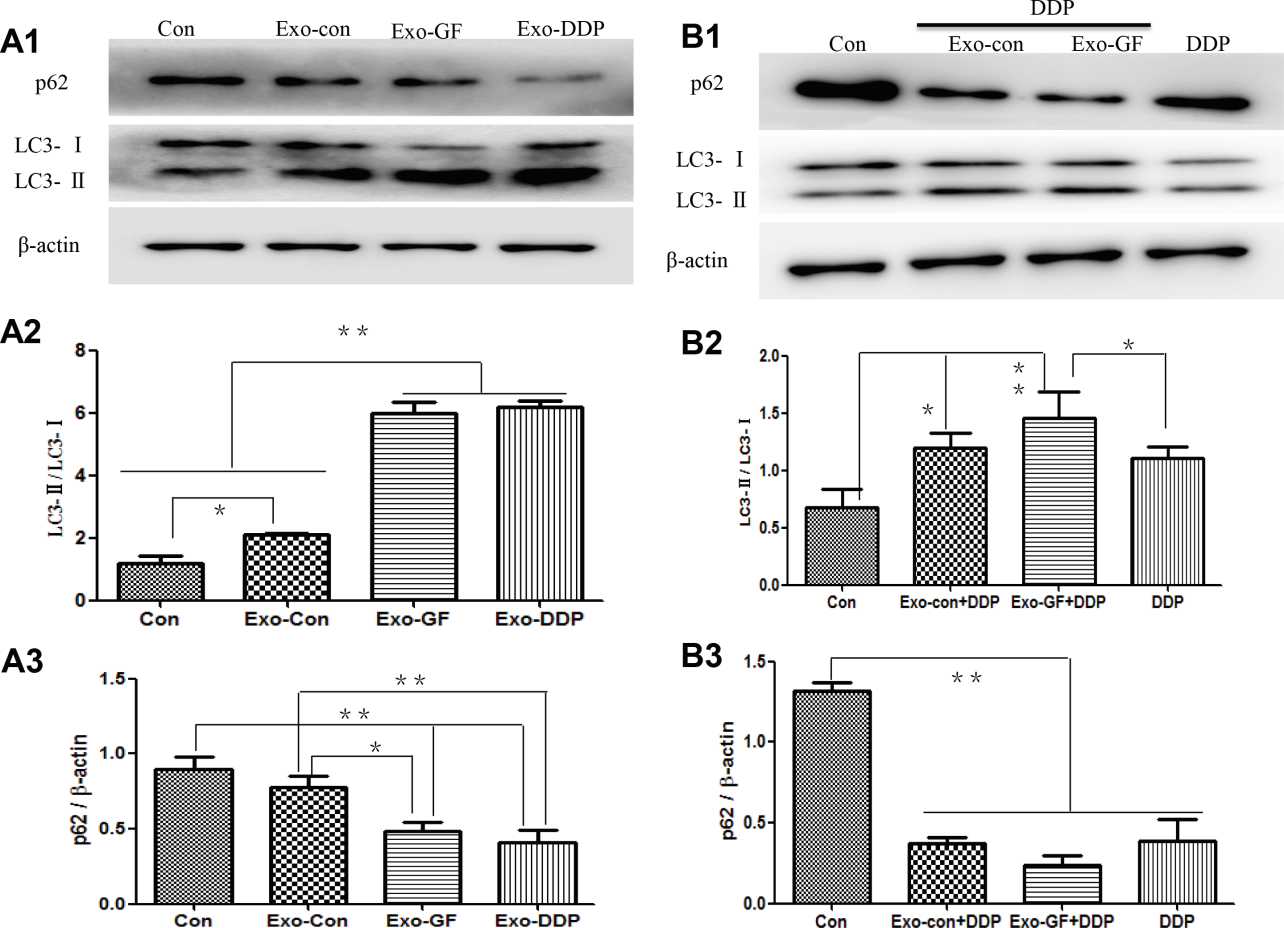
Exosomes upregulate autophagic activity and Exo-GF enhances cisplatin-induced autophagy in PC9 cells PC9 cells were co-cultured with 10 μg/ml exosomes derived from different disposals for 24 hours, and autophagic activity was investigated by western blot analysis (**A1**). LC3-II conversion (**A2**) and P62 degradation (**A3**) were analyzed using Scion Image software 4.0.3.2. The impact of Exo-GF on cisplatin-induced autophagy was also investigated by western blot analysis (**B1**) of LC3-II conversion (**B2**) and P62 degradation (**B3**) using Scion Image software 4.0.3.2. Data are presented as the mean ± SD (error bar) of at least three independent experiments. *and **represent *P* < 0.05 and *P* < 0.01, respectively.

To test whether Exo-GF could influence autophagic activity in cells, western blot analysis of LC3 conversion and p62 degradation was conducted. As shown in Figure 4A1, Exo-Con, Exo-GF and Exo-DDP could significantly up-regulate autophagy activity compared to the untreated PC9 cells. Exo-GF and Exo-DDP produced a greater increase in autophagic activity, as demonstrated by the semi-quantitative analysis of LC3 conversion (Figure 4A2) and p62 degradation (Figure 4A3). We further explored whether Exo-GF could enhance cisplatin-induced autophagy. As expected, Exo-GF co-cultured with cisplatin enhanced cisplatin-induced autophagy compared to the cisplatin-only group, as demonstrated by increased LC3 conversion and decreased p62 protein levels (Figure 4B1). Semi-quantitative analysis of LC3 conversion (Figure 4B2) and p62 levels (Figure 4B3) also confirmed that Exo-GF could increase cisplatin-induced autophagy (*p* < .05 *vs* DDP group). However, when Exo-DDP was co-cultured with gefitinib, this had no impact on gefitinib-induced autophagy (Figure S1).

### Exo-GF reduces cisplatin-induced apoptosis

**Figure 5:**
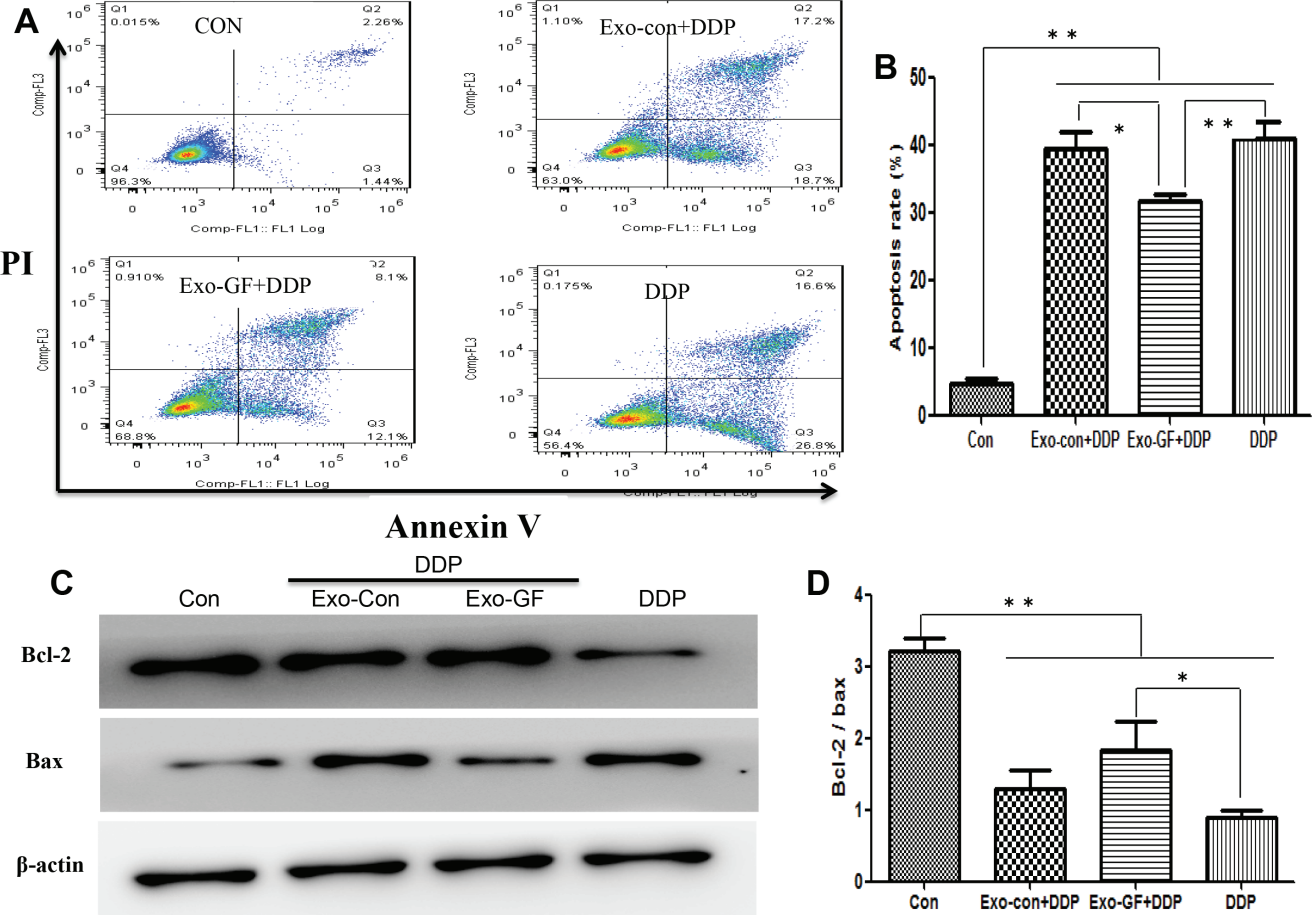
Exosomes derived from gefitinib-treated PC9 cells reduce cisplatin-induced apoptosis PC9 cells were pre-incubated with 10 μg/ml of Exo-Con or Exo-GF for 24 hours and co-cultured with 7.5 μM cisplatin for another 24 hours. Apoptotic cells were detected by FCM assay using an Annexin V-FITC/PI double-staining apoptosis detection kit (**A**) and statistically analyzed (**B**). The expression levels of Bcl2 and Bax protein were measured by western blotting (**C**) and analyzed using Scion Image software 4.0.3.2 (**D**). Data are presented as the mean ± SD (error bar) of at least three independent experiments. *and **represent *P* < 0.05 and *P* < 0.01, respectively.

We have previously reported that gefitinib in combination with cisplatin can induce cytoprotective autophagy and antagonize apoptosis. Thus, we investigated whether a reduction in apoptosis was mediated by exosomes. Flow cytometry (FCM) analysis (Figure 5A and 5B) revealed that co-incubation of Exo-GF with cisplatin could significantly reduce the number of apoptotic cells compared to either cisplatin alone or cisplatin co-incubated with Exo-Con. We also investigated whether Exo-DDP could affect apoptosis induced by gefitinib. Exo-DDP did not alter gefitinib-induced apoptosis (Figure S2).

To further confirm that the antagonistic effects were caused by a decrease in apoptosis, western blot assays were conducted to investigate the expression level of Bcl-2 and Bax. As expected, the protein levels of Bcl-2 were elevated and Bax levels were reduced after exposing PC9 cells to cisplatin co-incubated with Exo-GF (Figure 5C). The Bcl-2/Bax ratio is a common way to represent the degree of apoptosis. As shown in Figure 5D, the Bcl-2/Bax ratio in the untreated PC9 group was significantly higher than that of the cisplatin-contained group (*P* < .01 vs control group). The Bcl-2/Bax ratio was increased after Exo-GF was combined with cisplatin (*P* < .05 vs DDP group) compared to cisplatin alone. Exo-DDP did not alter the Bcl-2/Bax ratio after incubation with gefitinib compared to gefitinib alone (Figure S2).

## DISCUSSION

To our knowledge, this is the first report to reveal that exosomes contribute to the antagonistic effects of TKIs and chemotherapy agents in EGFR-mutated NSCLC cells. Exo-GF significantly reduced cisplatin-induced apoptosis and proliferation inhibition along with up-regulated autophagic activity. In contrast, Exo-DDP had no significant impact on gefitinib-induced apoptosis, proliferation inhibition and autophagy. Furthermore, inhibition of exosome secretion generated modest synergistic effects when GW4869 was co-administered with gefitinib and cisplatin. Thus, Exo-GF may play an important role in the antagonistic effects of gefitinib and cisplatin. Additionally, inhibition of exosome secretion may be a helpful strategy to reduce the antagonistic effects of gefitinib and cisplatin in EGFR-mutated NSCLC cells. Our results also suggest that administration of chemotherapy agents prior to TKIs, or after a washout period to completely eliminate TKI-related exosomes, could be a useful strategy to improve the therapeutic effects of chemotherapy agents.

Exosomes play a pivotal role in intercellular communication by releasing their contents, such as proteins, lipids and miRNA. There is evidence that exosomes are involved in modulating tumor cell proliferation, invasion and metastasis [13, 14]. However, to date, the precise role of exosomes on cell proliferation is unclear. Exosomes secreted by tumor cells are thought to package tumor-specific molecules, and promote tumor cell proliferation under various conditions, and this hypothesis has been verified by a number of studies [23, 24]. Furthermore, Xiao and colleagues reported that exosomes derived from DDP-treated A549 cells reduced the anti-proliferation effects of DDP in neighboring A549 cells [25]. This phenomenon was also observed in docetaxel-resistant breast cancer cells [26]. However, there is evidence of alternate mechanisms. For example, in a co-culture system of Huh7 cells, secreted exosomes significantly reduced recipient HepG2 cell growth and proliferation by transmitting miR-122 [27]. Meanwhile, exosomes isolated from doxorubicin or doxorubicin combined with heat-stress-treated MCF-7 cells significantly inhibited MCF-7 cell proliferation and triggered MCF-7 cell apoptosis [28]. This is consistent with our work, where we demonstrated that exosomes derived from PC9 cells treated with either gefitinib or cisplatin could modestly inhibit proliferation of co-cultured PC9 cells (data not shown).

Resistance mediated by transmitting exosomes has been observed in several tumors, such as ovarian, prostate and NSCLC [25, 29, 30]; however, less is known about exosomes and the antagonism of TKIs and chemotherapy agents. In the present study, proliferation inhibition of PC9 cells by cisplatin was reduced in a dose-dependent manner in the presence of Exo-GF. However, Exo-DDP had no impact on gefitinib, which suggests that Exo-GF may counteract the antitumor activity of cisplatin. Inhibition of exosome secretion by GW4869 increased the growth inhibition ratio induced by co-administration of gefitinib and cisplatin, but CDI analysis indicated a modest synergistic effect. The CDI values were significantly different in the presence or absence of GW4869, suggesting that inhibition of exosome secretion could overcome the antagonistic effects between gefitinib and cisplatin. However, the exact mechanism by which exosomes mediate resistance is still unclear. Indeed, several studies have reported that exosomes may alter the drug sensitivity of recipient cells in various cancer models via transferring miRNAs or ABC proteins [29, 31].

We have previously reported that up-regulation of autophagy contributed to the antagonistic effects between gefitinib and cisplatin [19]; thus, in the present study, we investigated whether up-regulated autophagy was mediated by exosome secretion. Previous studies using co-culture systems of breast cancer cell lines and fibroblasts have shown that ROS are generated and induce autophagy in tumor-associated fibroblasts [32]. Furthermore, Dutta et al. [33] demonstrated that exosomes derived from breast cancer cells could induce autophagy in HMECs. However, the relationship between exosomes and autophagy in the antagonism of TKIs and platinum-based chemotherapy has not been studied. In the present study, we found that Exo-Con, Exo-GF and Exo-DDP could all up-regulate autophagy. However, either Exo-GF or Exo-DDP induced significantly higher autophagic activation compared to Exo-Con or control cultured PC9 cells, which suggests that gefitinib and cisplatin may transmit autophagy signals to neighboring or distant cells via exosome secretion.

Interestingly, Exo-GF co-incubation with cisplatin increased autophagic activity induced by cisplatin, as demonstrated by the increased LC3 conversion and decreased p62 protein expression. However, Exo-DDP co-incubation did not increase gefitinib-induced autophagic activity. The underlying mechanisms of this phenomenon are not clear, but it is possible that gefitinib alone could induce a higher level of autophagy than cisplatin, which was observed in our previously studies [19], and it is sufficient for survival in poor environments; thus, there is no need for further up-regulation of autophagic activities. In the present study, the autophagic activities induced by cisplatin were modest when compared to gefitinib-induced autophagy, so when co-administration of cisplatin with Exo-GF cells up-regulates autophagy to maintain homeostasis.

Our results also help to illustrate the phenomenon that the co-administration of cisplatin and TKIs results in antagonism, while sequential administration of cisplatin and TKIs produce a synergistic effect. Following co-administration of cisplatin and gefitinib, exosomes released by gefitinib-treated cells up-regulated autophagic activity in uninfluenced cells and up-regulated autophagy to counteract cisplatin-induced apoptosis. However, following sequential administration of chemotherapy agents and TKIs, exosomes induced by TKIs might have been removed or metabolized at a relatively low level due to several days of washouts, thus preventing cytoprotective autophagy. Thus, the administration of TKIs after chemotherapy and a washout period before the next cycle of chemotherapy may be a better strategy in clinical settings.

## CONCLUSIONS

In this study, we observed that Exo-GF could counteract apoptosis induced by cisplatin by up-regulating autophagy. Inhibition of exosome secretion by GW4869 could reverse the antagonistic effects of gefitinib and cisplatin, at least in part, which suggests that inhibition of exosome release might be a feasible and promising strategy for lung cancer treatment. Furthermore, our observations also suggest that sequential administration of chemotherapy after a washout period of EGFR-TKIs before the next cycle of chemotherapy may be a better protocol for clinical applications.

## MATERIALS AND METHODS

### Cell culture and reagents

The human NSCLC cell lines PC9 and A549 (Cell Bank of the Chinese Academy of Sciences, Shanghai, China) were maintained in RPMI 1640 medium (Gibco, USA) and supplemented with 10% exosome-depleted fetal bovine serum (System Biosciences, USA).

Gefitinib (Santa Cruz, USA) and cisplatin (Qilu pharmaceutical, China) were re-suspended in dimethyl sulfoxide (DMSO) and phosphate buffer solution (PBS), respectively, and stocked at a concentration of 10 mM at −20°C. The anti-LC3 and anti-P62 antibodies as well as GW4869 were purchased from Sigma (Sigma-Aldrich, USA). Anti-Bcl-2 and anti-Bax antibodies were purchased from Cell Signaling Technology (CST, USA). An Exoquick-TC^™^ exosome isolation kit and anti-CD63 antibody were purchased from SBI (System Bioscience, USA). Anti-β−actin and anti-rabbit and anti-mouse IgG peroxidase-conjugated secondary antibodies were obtained from Bioworld (USA). A cell counting kit-8 (CCK-8) was purchased from Bestbio (Bestbio, China). A BCA protein assay kit was purchased from Beyotime (Beyotime, China). Cell apoptosis was measured using an Annexin V-FITC/propidium iodide (Bestbio, China) double-staining assay and flow cytometry (Cytomics^™^ FC 500, Beckman Coulter, USA).

### Exosome isolation and characterization

Exosomes were obtained from the cell supernatant of untreated (Con) cultured PC9 cells or cells treated with gefitinib (1 μM) or cisplatin (7.5 μM) using ExoQuick Precipitation Solution as described previously [34]. The exosomes were designated as Exo-Con, Exo-GF (gefitinib) and Exo-DDP (cisplatin) for simplicity. Briefly, cell supernatants were harvested and centrifuged at 3000 × g for 15 minutes to remove cell debris. Next, supernatants were transferred to another sterile vessel, and 0.2 ml of the ExoQuick precipitation solution was added to 1 ml of cell supernatant and incubated overnight at 4°C. Then, the mixture was centrifuged at 1500 × g for 30 minutes; the supernatant was discarded, and the pellet was centrifuged at 1500 × g again for 5 minutes. Excess fluid was removed. The final pellets were resuspended in 200 μl of PBS and stored at −80°C. The exosomal protein concentrations were quantified and standardized using a BCA protein assay kit.

### Transmission electron microscopy

TEM was performed as previously reported to demonstrate exosome formation [35]. Briefly, exosomes were isolated and diluted in 100 μl of PBS, and 20 μl of the suspension was placed onto formvar carbon-coated copper grids at room temperature for 1 minute. The excess suspension was removed using filter paper. Exosomes were stained by 2% phosphotungstic acid (PTA) at room temperature for 5 minutes. The grids were then fixed with 2% glutaraldehyde at room temperature for 5 minutes, followed by rinsing with PBS three times. Images were obtained under 80 kV with a Jeol JEM-1010 (Tokyo) transmission electron microscope (JEM-1230; Jeol Ltd., Japan).

### Cell viability assays

The interaction between gefitinib and cisplatin was tested using the same methods we have reported previously [19]. To explore the impact of exosomes on the anti-proliferation of gefitinib or cisplatin in PC9 cells, exosomes were incubated for 24 hours, followed by the addition of 1 μM gefitinib or 7.5 μM cisplatin. After an additional 24-hour incubation, 10 μl of CCK-8 agent was added to each well and incubated for 2 hours at 37°C. The optical density (OD) values were read on an enzyme-labeled ELx800 (Bio-Tek, USA) at 450 nm. The cellular proliferation inhibition rate (IR) was calculated according to the following equation: IR = [1–(average OD value of experimental group)/(average OD value of control group)] × 100%.

### Analysis of *in vitro* drug interaction

The coefficient of drug interaction (CDI) is widely used to assess the synergistic or antagonistic effects of drug combinations. CDI was employed to assess the nature of drug combinations, as reported previously [19, 36]. Briefly, CDI was measured according to the absorbance of each group and calculated using the following equation: CDI = AB/(A × B). AB is the ratio of the OD values of the combination groups divided by control group, while A and B are the ratios of the OD values of single agents divided by the control group. A CDI value of less than 0.95, greater than 1.05 or between 0.95 and 1.05, indicates synergistic, antagonistic and additive effects, respectively.

### Flow cytometry

Apoptosis was detected using flow cytometry (FCM) with an Annexin V-FITC/PI double-staining kit. Briefly, cells were plated and treated for use in the cell viability assay. Following incubation for 24 h, the supernatant and attached cells were collected and stained with 10 μl of Annexin V-FITC and 5 μl of PI at 4°C for 20 minutes in the dark and analyzed using a flow cytometry system (Beckman Coulter, Fullerton, CA, USA).

### Western blot analysis

Western blot analysis was performed as previously described [19]. Briefly, cells and exosomal lysates were centrifuged at 12, 000 × g for 10 minutes at 4°C, and the supernatants were resolved. Approximately 20 μg of total protein was loaded and electrophoresed via 12.5% SDS-PAGE and subsequently transferred onto nitrocellulose membranes. Proteins were detected by incubation with an indicated antibody at 4°C overnight. The immunoreactive bands were visualized using the enhanced chemiluminescence reagent (Thermo Fisher, USA), and signals were captured using an Image Quant^™^ LAS-4000 Mini Imager (Fuji, Japan). For semi-quantitative analysis, the protein density of each band was determined using Scion Image Software, version 4.0.3.2.

### Statistical analysis

SPSS 16.0 software (SPSS Inc., Chicago, USA) was used for statistical analyses. A two-tailed Student's *t*-test was applied to evaluate the difference between two groups, and one-way ANOVA with multiple comparisons was employed for comparing three or more groups. All experiments were independently carried out in triplicate, and the results were presented as the mean ± standard deviation (SD). Differences were considered to be statistically significant with a *p*-value of less than 0.05.

## SUPPLEMENTARY MATERIALS FIGURES


