# Supplementary material for: Exosomes derived from gefitinib-treated EGFR-mutant lung cancer cells alter cisplatin sensitivity via up-regulating autophagy
Source: Oncotarget. 2016 Mar 25;7(17):24585–95. doi: 10.18632/oncotarget.8358 (PMC5029725; doi:10.18632/oncotarget.8358)
Supplement: Supplementary file 1 [file oncotarget-07-24585-s001.pdf]

Exosomes derived from gefitinib-treated EGFR-mutant lung cancer cells alter cisplatin sensitivity via up-regulating autophagy

Supplementary Materials

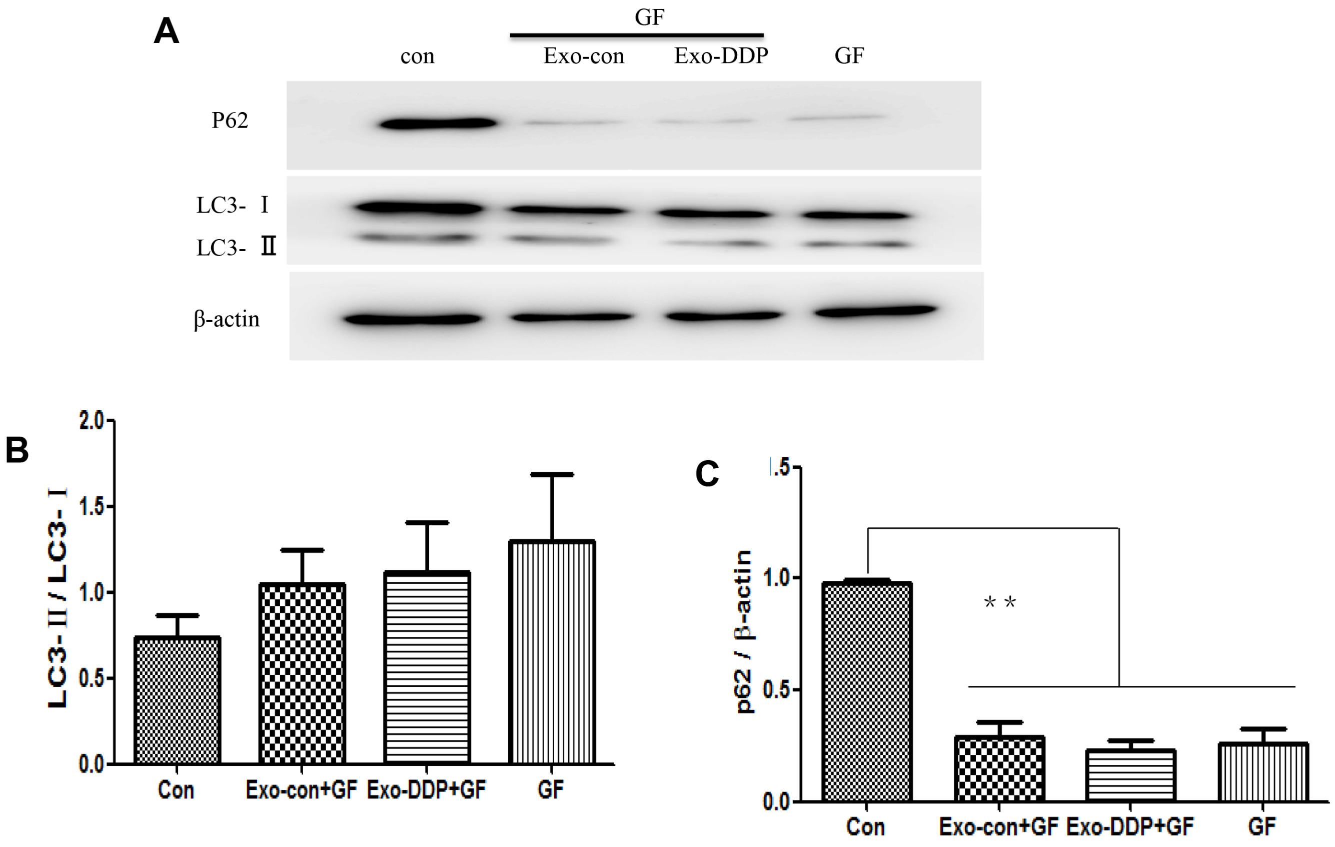

Supplementary Figure S1: Exo-DDP did not significantly impact gefitinib-induced autophagy. The impact of Exo-DDP on gefitinib-induced autophagy was investigated using western blots (**A**), semi-quantitative analysis of LC3-II conversion (**B**) and P62 degradation (**C**) using Scion Image software 4.0.3.2. Data are presented as the mean ± SD (error bar) of at least three independent experiments. *and **represent $P < 0.05$ and $P < 0.01$, respectively.

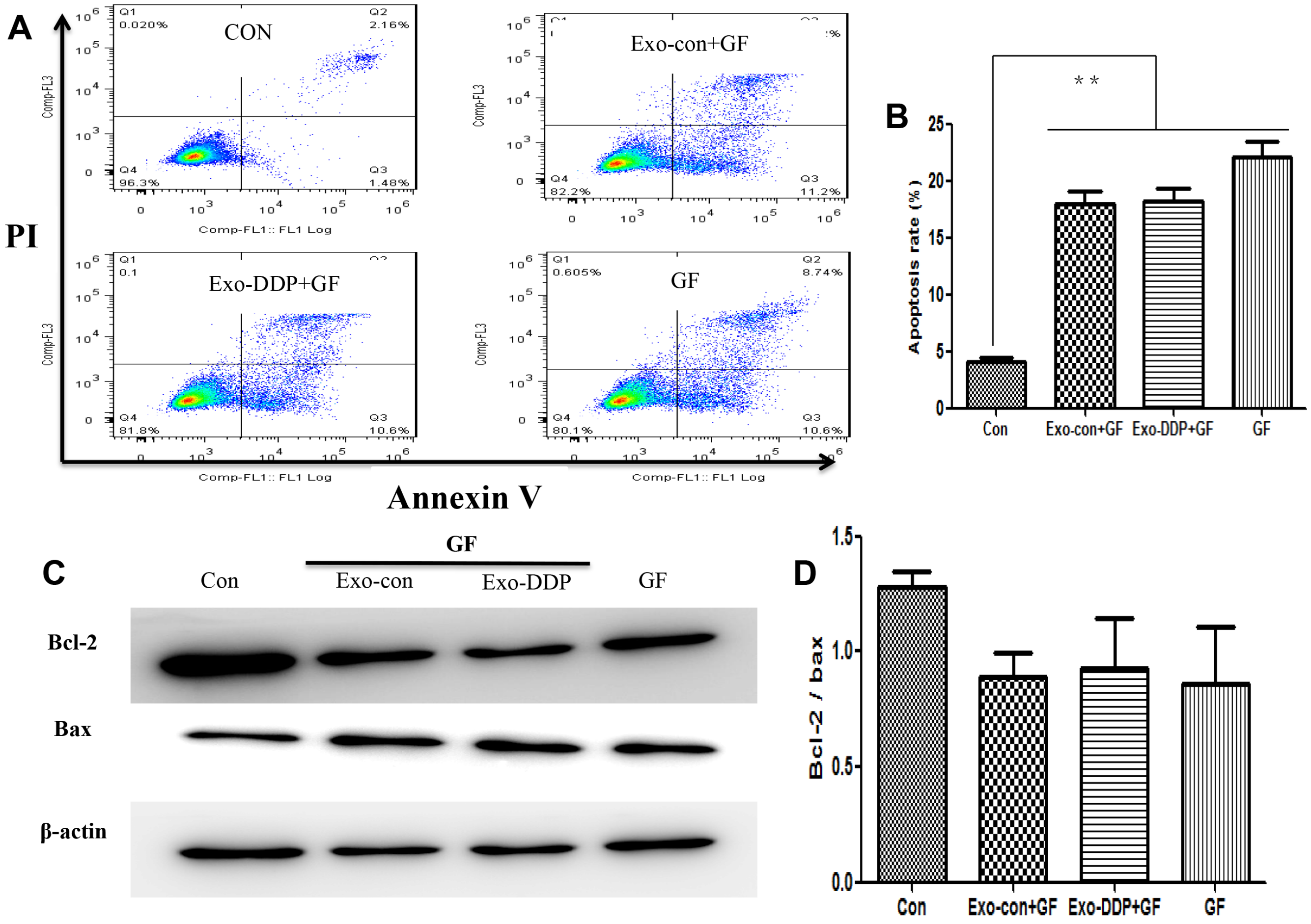

Supplementary Figure S2: Exosomes derived from cisplatin-treated PC9 cells did not significantly impact gefitinib-induced apoptosis. PC9 cells were pre-incubated with 10 µg/ml of Exo-Con or Exo-DDP for 24 hours and co-cultured with 1 µM gefitinib for another 24 hours. Apoptotic cells were detected by FCM assay using an Annexin V-FITC/PI double-staining apoptosis detection kit (**A**) and statistically analyzed (**B**). The expression levels of Bcl2 and Bax proteins were measured by western blotting (**C**) and semi-quantified using Scion Image software 4.0.3.2 (**D**). Data are presented as the mean ± SD (error bar) of at least three independent experiments. **represents $P < 0.01$ for the indicated groups.
